# Rush Medical College

**Published:** 1845-01

**Authors:** J. H. Bird


### ILLINOIS

### MEDICAL & SURGICAL JOURNAL.

VOL. I. JANUARY, 1845. NO. 10.

### RUSH MEDICAL COLLEGE.

### PROF. BRAINARD'S SURGICAL CLINIC. DEC. 2d, 1844.

[Reported for the Journal, by J. H. BIRD.]

CASE 1.—Mr. ——— having a disease of the eye, was presented. Dr. Brainard remarked that this man has a violent disease of the eye; he suffered, according to his own account, with some acute inflammation of an internal organ, to which succeeded severe pain in the eye and great inflammation. He has inflammation of the conjunctiva with onyx, the result of inflammation of the cornea. Onyx is the collection of pus within the lamellæ of the cornea. It may discharge itself externally, causing an opacity with staphyloma, or protrusion of its surface. In inflammation of the cornea, you may observe a vascular zone on the sclerotica sourrounding the cornea, the redness of which, if inflammation of the conjunctina is absent, is less marked at a distance from the cornea. A good diagnostic sign in diseases of the eye is, that in inflammation of the cornea, sclerotica or the inner parts of the eye, the patient has a great dread of light—not so in inflammation of the conjunctiva.

The object to be effected, is the removal, as far as possible, of the opacity of the cornea, and of the granulated state of the conjunctiva. The removal of the former is best effected by the action of mercurials; the anti-plastic and absorbent power of the medicine having good effect upon the disease. We will remove the thickened state of the conjunctiva, by a solution of nitrate of silver, grs. ij. to the ounce of water, one or two minims dropped into the eye several times a day—with regular regimen and diet

—with the administration of the following prescription:—℞. Calomel grs. iij., opium gr. ¼ every night until system is affected, a cure may be expected.

---

Case 2.—Mr. S., ætas 20, having converging strabismus of right eye, presented himself for operation. Dr. B. remarked that the operation for the cure of strabismus was first performed by Dieffenbach, in 1840, and like all new discoveries, it had numerous admirers, and surgeons who could not number their operations, by hundreds of cases, were ashamed of their inactivity; but the effects of the operation not equalling the reports of sanguine friends, or the expectations of patients, it fell into disrepute, and surgeons now are not as anxious to operate as formerly. Strabismus is a deviation of the axis of the eye from its true position; the true axes of the eyes are slightly convergent. Strabismus may be either converging or diverging. There is a variety in children from spasmodic action of the muscles, from cerebral irritation, generally converging, which spontaneously disappears after the removal of cause, or requires operation. Rheumatic inflammation of the tendons produces quite often their contraction, so rheumatic inflammation of the eye, or its muscles, may produce the deformity under notice. Diverging strabismus may be caused by a paralysis of the third pair of nerves; numerous other causes may produce the disease, among which is the difference of power in the two eyes, causing greater use of the sound one. The division of the contracted tendon or tendons is the remedy indicated The point of division is either near the margin of the sclerotica, or farther removed; I prefer the first, as in the latter, owing to the distance between tne conjunctiva and tendon, granulations may arise from the surface of the incision, which require generally removal by excision. The tendons of the recti muscles are expanded into a continuous fascia, which surrounds the globe of the eye and gives that pearly appearance to the sclerotica, seen beneath the thin membrane, the conjunctiva, covering it. The want of success in the operation often results from not sufficiently separating the attachments of this fascia to the sclerotica. A bad effect of the operation, when but one eye has been operated upon, is sometimes a staring appearance; if both eyes are operated upon they are uniform, therefore an operation upon both, if practicable, is preferable. As a remedy for this staring, I propose the division of the tendon of the levator palpabræ superioris, or of the expansion sent from the superior rectus to the upper lid, which will enable the lid to fall more easily over the eye. The dressings, after the operation, are a simple compress and roller; the period of confinement should be from nine to twelve days. The present deformity has existed from infancy, and is ascribed to hooping cough.

The Doctor described the various instruments used in the operation, but preferred the forceps for retaining the eye in its position, to the double hooks recommended by authors, and stated that he generally divided the conjunctiva to the extent of three-fourths of an inch. The operation was performed and the patient dismissed.

---

Case 3.—Mr. ——— having inflammation of the eye.

*Remarks.*—This is a case of inflammation of the conjunctiva of a year's duration, now become chronic. There was originally conjunctivitis, but the cornea has also been inflamed, and the eye presents the following appearances: thickening of the conjunctiva covering both the lids and eye, dullness of the cornea, it having lost its polish, with irregularity of its surface. The disease is too far advanced to remove the nebulosity of the cornea, and it will be permanent. You will often find patients complaining of weakness of the eye, which results from a chronic inflammation of the conjunctiva with slight granulation. There is also generally connected with it, inflammation of the meibomian glands, with a change in their secretions, from a healthy fluid which lubricates the margins of the lids, to a puriform one, producing an adherence of them. The ointment of the nitrate of mercury, in the proportion of one part to four of lard, or ℞.—Sulph. zinc vj., spermaceti oint. ℥j., form good applications in the latter disease.

In private practice, the necessary rules of regimen, diet, &c., cannot be enforced as in hospital practice, so the cure is not as certain or as speedy. I employ alternate scarifications and cauterizations. As a cautery I use generally the sulphate of copper in substance, applied lightly to the surface, or the solid nitrate of silver. The patient has used various empirical remedies, Thompson's eye water, which is a combination of acetate of lead and opium, and other irritants, until I wonder he has any eyes at all. As bleeding and purging are not necessary in this case, we will merely prescribe the ointment mentioned, and alternate scarifications and cauterizations.

CASE 4.—Miss ———, ætas 19, with converging strabismus of right eye and slight convergence of left.

*Remarks.*—In regard to the success of the operation. This is my forty-second case, and at least in three-fourths of the number operated upon, the result has been quite successful. As I sufficiently enlarged upon the subject of strabismus in a previous lecture, I will merely mention that the indication of the deformity in both eyes, is judged of by the extent of lateral movements of the eyes, and the extent the cornea may be hid by the internal commisure of the eyelids. The Dr. then performed the operation and the patient dismissed. A few days after, the operation was found to have been successful, the eye having taken its true position and the incision having healed.

---

CASE. 5.—Mr. ——— having disease of the eye.

*Remarks.*—Gentlemen: To illustrate the subjects already treated of, I bring before you a case of traumatic inflammation of the eye, caused by a blow upon the eye. There is a wound of the lower lid, haziness of the cornea, and opacity at its internal parts. There is chronic inflammation of the cornea, you may observe the vascular zone around its margin; there is a slight inflammation of the conjunctiva; there is also inflammation of the iris, the pupil is contracted and deformed, and not at the centre of the cornea; there is a marked difference in the color of the irides, the affected one of a darker and reddish hue. These morbid appearances are the result of a blow from a board, about three weeks since. The patient experienced much pain, seated in the orbit and extending upwards upon the forehead, in the temporal region, and upon the face and nose; which was more violent at night, as is the case in inflammation of the fibrous tissue. The treatment indicated is to remove the inflammation and restore, as far as possible, the eye to its normal state. The effects of our treatment will leave the cornea clear, the pupil immoveable; there will be opacity of the lens. The restoration of sight will be impossible, but by means of our treatment, the eye will be left in such a state, that by the operation for cataract, the patient can see, if the other eye should be lost, which is liable to occur, owing to the great sympathy existing between them. Conjunctivitis, as I have said before, is not benefitted by the use of mercurials, but in inflammation of the cornea, these are of essential benefit. We will therefore prescribe calomel, grs. iij., three times per day until system is slightly af ected; also the application to the eye of ℞.—Belladonna grs. iv. to water f℥j., to dilate the pupil; and make a stimulant application of ℞.—Nitrate silver grs. iij., water f℥j., in the usual manner. A blister to the nuchæ would also be serviceable.

---

Case 6.—Mr. ——— with chronic conjunctivitis.

*Remarks.*—This case presents all the ordinary appearances of the chronic inflammation of the conjunctiva, granulation, pannus, opacity of the cornea, and blepharitis, which you will meet with quite often in your practice. This case is the result of active conjunctivitis, three years since, and now the disease has become chronic. Our treatment is alternate scarifications and cauterizations with sulphate of copper, and the application of citrine ointment to the lids. This treatment is usually successful, if properly pursued, but patients generally object to the confinement; a period from three to six months would restore the eye to its healthy condition; but a few weeks will show a decided improvement. As our patient consents to some confinement, you will have the opportunity of judging for yourselves. Purging and depletion are not admissible, but a guarded regimen and a good nutritious diet is to be advised.

